# Single-cell and bulk RNA-sequencing reveal PRRX2-driven cancer-associated fibroblast-mediated perineural invasion for predicting the immunotherapy outcome in colorectal cancer

**DOI:** 10.3389/fcell.2025.1620388

**Published:** 2025-09-29

**Authors:** Mingxiao Chen, Yue Cai, Feng Han, Bo Li, Zhou Xu, Kaili Cui, Wenqi Bai, Feng Li

**Affiliations:** ^1^ Department of Radiation Oncology, Shanxi Province Cancer Hospital/Shanxi Hospital Affiliated to Cancer Hospital, Chinese Academy of Medical Sciences/Cancer Hospital Affiliated to Shanxi Medical University, Taiyuan, China; ^2^ Department of Anesthesiology, Shanxi Province Cancer Hospital/Shanxi Hospital Affiliated to Cancer Hospital, Chinese Academy of Medical Sciences/Cancer Hospital Affiliated to Shanxi Medical University, Taiyuan, China; ^3^ Department of Information Management, Shanxi Province Cancer Hospital/Shanxi Hospital Affiliated to Cancer Hospital, Chinese Academy of Medical Sciences/Cancer Hospital Affiliated to Shanxi Medical University, Taiyuan, China; ^4^ Department of Gastroenterology, Shanxi Province Cancer Hospital/Shanxi Hospital Affiliated to Cancer Hospital, Chinese Academy of Medical Sciences/Cancer Hospital Affiliated to Shanxi Medical University, Taiyuan, China; ^5^ Central Laboratory, Shanxi Province Cancer Hospital/Shanxi Hospital Affiliated to Cancer Hospital, Chinese Academy of Medical Sciences/Cancer Hospital Affiliated to Shanxi Medical University, Taiyuan, China; ^6^ Department of Colorectal Surgery, Shanxi Province Cancer Hospital/Shanxi Hospital Affiliated to Cancer Hospital, Chinese Academy of Medical Sciences/Cancer Hospital Affiliated to Shanxi Medical University, Taiyuan, China

**Keywords:** PRRX2, perineural invasion, colorectal cancer, single-cell RNA, immunotherapy

## Abstract

**Background:**

Perineural invasion (PNI) is common in a variety of solid tumors and has been identified as an important pathway promoting tumor local invasion and distant metastasis. Its presence is usually associated with increased aggressiveness, malignant biology, and a worse patient prognosis. However, its specific role and regulatory mechanisms in colorectal cancer (CRC) remain unclear.

**Methods:**

In this study, we integrated 20 CRC single-cell transcriptome datasets, which contained 575,768 high-quality cells, and used the Scissor algorithm to map PNI phenotypes in TCGA bulk samples to the single-cell level. Nine cancer-associated fibroblast (CAF) subpopulations were identified and functionally annotated. We evaluated the clinical relevance of CAF subsets in TCGA and three independent cohorts (silu_2022, GSE39582, and GSE17536) using BayesPrism-based deconvolution. We analyzed transcriptional regulatory networks using pySCENIC and validated PRRX2 function by *in vitro* experiments. Immune infiltration characteristics were quantified using the ssGSEA score, and the association between the PRRX2 score and immune checkpoint inhibitor efficacy was analyzed in conjunction with two immunotherapy cohorts. In addition, we performed a drug sensitivity analysis based on the GDSC pharmacogenomics database to screen potential therapeutic agents.

**Results:**

In this study, we systematically revealed the characteristics of the perineural invasion-associated fibroblast subsets and their regulatory mechanisms. In PNI-positive tumors, the proportion of fibroblasts was significantly increased, with the enrichment of MMP2+ myofibroblastic cancer-associated fibroblasts (myCAFs), and facilitated perineural infiltration through extracellular matrix remodeling. Further analysis revealed that PRRX2 was a core regulator of MMP2+myCAFs, promoting perineural invasion through the activation of TGF-β signaling pathways. PRRX2 knockdown significantly inhibited fibroblast proliferation, clonogenic formation, and invasive migration capacity, and it reduced TGFB1 and NGF expressions. The clinical cohort validation demonstrated a significant correlation between the PRRX2-score and advanced tumor stage, along with vascular and lympho-vascular invasion (LVI). Furthermore, patients with high PRRX2 scores had a significantly worse prognosis. In addition, patients with high PRRX2 scores responded poorly to immune checkpoint inhibitors but may be sensitive to targeted agents or antibody-coupled drugs, which may serve as potential targets for combination therapy.

**Conclusion:**

This analysis established PRRX2-driven MMP2+myCAFs as pivotal mediators of CRC perineural invasion through TGF-β/ECM remodeling. The PRRX2 score serves as a biomarker for prognosis prediction and immunotherapy outcome.

## 1 Introduction

Colorectal cancer (CRC) is the third most prevalent malignant gastrointestinal neoplasm tumor worldwide, with nearly 20% recurrence within 5 years (Qaderi et al., 2021; Wang et al., 2024). In recent years, perineural invasion (PNI) has emerged as an independent metastatic pathway in a variety of gastrointestinal neoplasms (Que et al., 2024; Li et al., 2021; Yang et al., 2020). Several studies have shown that PNI is associated with poor survival prognosis in gastric cancer, pancreatic cancer, and other tumors, and is often used as an essential basis for early treatment decisions (Cienfuegos et al., 2017; Kang et al., 2022). In CRC, the incidence of PNI ranges from 20% to 30% (Li et al., 2022). However, the molecular mechanism of PNI in patients with CRC remains unclear. Understanding the underlying mechanisms of PNI and exploring potential predictive biomarkers may aid in more precise clinical management.

Recent studies have shown that PNI has made significant advances in exploring mechanisms and functions across various cancers. Cancer-associated fibroblasts (CAFs) have been identified as a key factor in promoting PNI events. Previous studies have demonstrated that CAFs facilitate PNI by secreting extracellular vesicles (EVs) to carry PNI-associated transcripts in pancreatic cancer (Zheng et al., 2024). In oral squamous cell carcinomas, CAFs can secrete factors such as CXCL12 and IL-6 to activate Schwann cells and induce the expression of neurotrophic factors and axon guidance-related proteins, leading to enhanced neural infiltration of tumor cells (Zhang et al., 2025). A high level of CAF infiltration is associated with advanced TNM stages and a more severe degree of PNI in gastric cancer (Zhang J. et al., 2020). However, CAFs are highly heterogeneous (Luo et al., 2022). Hence, an in-depth study of CAFs may aid in understanding the mechanism and function of PNI.

In this study, we integrated 20 single-cell RNA sequencing (scRNA-seq) datasets with 575,768 cells from 291 patients. MMP2+myCAFs were enriched in the PNI+ group and positively associated with advanced clinical stages and poor prognosis. PRRX2 was identified as a key transcriptional regulator of MMP2+myCAFs using the pySCENIC analysis. Based on PRRX2 and its target genes, we developed a PRRX2-score risk prediction model. We found that the PRRX2 score was associated with multiple clinical information and predicted prognosis. The PRRX2 score could predict the benefit of immunotherapy. Patients with low PRRX2 scores responded to immunotherapy, whereas those with high PRRX2 scores had a poor prognosis. However, through drug sensitivity analysis, we found that patients with a high PRRX2 score could potentially benefit from targeted drugs (ZM447439, BMS.754807, etc.), ADCs (PIK3CA, MUC1, MET, etc.), and other combinations of treatment (CZC24832, KU.55933, staurosporine, etc.).

## 2 Methods

### 2.1 Data collection

In this study, we integrated 20 scRNA-seq datasets of CRC, encompassing 291 patients. These datasets were obtained from the Gene Expression Omnibus (GEO) database (https://www.ncbi.nlm.nih.gov/geo/) and EMBL-EBI ArrayExpress (https://www.ebi.ac.uk/arrayexpress/), with the following accession numbers: GSE132257 (Lee et al., 2020), GSE132465 (Lee et al., 2020), GSE146771 (Zhang L. et al., 2020), GSE166555 (Uhlitz et al., 2021), GSE178341 (Pelka et al., 2021), GSE188711 (Guo et al., 2022), GSE200997 (Khaliq et al., 2022), GSE201349 (Becker et al., 2022), GSE205506 (Zhou et al., 2024), GSE231559 (Hsu et al., 2023), GSE178318 (Che et al., 2021), GSE161277 (Zheng et al., 2022), GSE179784 (Sui et al., 2022), GSE236581 (Chen et al., 2024), GSE242271, GSE245552 (Liu et al., 2024), GSE163974 (Wang et al., 2021), GSE234804 (Berlin et al., 2023), GSE144735, and E-MTAB-8107. Additionally, we incorporated multiple bulk RNA-seq datasets, including The Cancer Genome Atlas (TCGA) COAD and READ project (TCGA cohort) from cBioPortal, the Siluo et al. cohort (silu_2022 cohort) (Roelands et al., 2023), and the GEO datasets GSE39582 (Marisa et al., 2013) and GSE17536 (Smith et al., 2010) (Supplementary Tables S1–S3). Immunotherapy-related data were derived from the following cohorts’ enrolled patients treated with immune checkpoint inhibitors: the Korean gastric cancer cohort (Kim et al., 2018) and the IMmotion151 study, a phase-III randomized controlled trial evaluating atezolizumab plus bevacizumab versus sunitinib in advanced renal cell carcinoma (Motzer et al., 2022). PNI annotations were extracted from postoperative pathological reports provided by TCGA, which were accessed via the cBioPortal platform (Gao et al., 2013). For the TCGA cohort, clinical data, survival outcomes, and bulk transcriptomic profiles were retrieved from the National Cancer Institute’s GDC Data Portal. Clinical and prognostic information for the GSE39582 and GSE17536 cohorts was sourced from its original publication (Kim et al., 2018).

### 2.2 Data processing and analysis

The following analysis was performed using Seurat R package (v 4.4.0) (Hao et al., 2021). The NormalizeData function normalized raw counts relative to the total library size. The top 2,000 highly variable features were identified by the FindVariableFeatures function. Focusing on these genes was helpful for highlighting biological signals in downstream analysis. The ScaleData function was utilized to scale the data. After using RunPCA for dimension reduction, harmony (v 1.2.3) was employed to eliminate batch effects between samples. Then, the RunUMAP function was applied for visualization with the top 30 harmony-reduced principal components. The UMAP plots before and after batching are shown in Supplementary Figures S1A, B. To evaluate clustering consistency after integration, we calculated silhouette scores based on the harmony-reduced embedding. To reduce computational complexity in this large dataset, we implemented a subsampling strategy by randomly selecting up to 5,000 cells per cluster. Silhouette widths were computed using the silhouette () function from the cluster R package, thus measuring intra-cluster cohesion and inter-cluster separation. Violin plots of silhouette distributions across major cell types are shown in Supplementary Figure S1C. FindNeighbors and FindClusters functions were used to cluster different cells with a resolution of 0.5. Ultimately, UMAP was applied to display the clustering results in a two-dimensional space. Subsequently, we annotated each cluster to specific cell types using known classical canonical markers. The following genes were utilized for cell-type annotation: fibroblasts (FAP and COL1A1), epithelial cells (EPCAM and KRT19), endothelial cells (VWF and PECAM1), B cells (CD19, MS4A1, and MZB1), myeloid cells (FCGR3A and CD68), T cells (CD3D and CD3E), and mast cells (TPSAB1 and CPA3). Subpopulations of fibroblasts were annotated, and each subpopulation was labeled according to its marker gene (Supplementary Table S4). Differences in gene expression between the subpopulations were analyzed by the “FindAllMarkers” function using the following parameters: min.pct = 0.1, logfc.threshold = 0.25, and only.pos = T. False discovery rate (FDR) correction was automatically applied within the FindAllMarkers function using the Benjamini–Hochberg (BH) method, and adjusted p-values (p_val_adj) were used to determine statistical significance. Pathway analysis was performed for each subpopulation using the Kyoto Encyclopedia of Genes and Genomes (KEGG) database with the R package clusterProfiler (v4.12.1) with BH FDR correction (Yu et al., 2012). Pathways with adjusted p-values (p.adjust) <0.05 were considered to be statistically significant.

### 2.3 Assessment of cell-type infiltration based on deconvolution for bulk RNA datasets

To assess the function of different cell types in bulk RNA datasets, we employed the Bayesian approach known as the BayesPrism (v 2.2.2) method, which jointly predicts the cellular composition and gene expression for each cell type (Chu et al., 2022). Based on the single-cell read count matrix, we constructed cell-type-specific expression profiles and estimated the proportions of cell types from public large-scale RNA-seq using the constructed cell-type reference. Differences in the infiltration proportions of specific subpopulations across distinct clinicopathological groups were assessed using a two-sided Wilcoxon rank-sum test, followed by multiple testing correction using the Holm method. Furthermore, the infiltration proportions of the subpopulations were also used to assess prognosis in bulk cohorts to evaluate prognosis.

### 2.4 Transcription factor regulator analysis

Regulatory networks and regulator activities were analyzed using the pySCENIC framework with default parameters (version 0.11.2) (Van de Sande et al., 2020). Specifically, subgroup-specific transcription factors (TFs) were identified through the Wilcoxon rank-sum test. Additionally, the Jensen–Shannon divergence was calculated using the philentropy software package, which provided the regulon specificity scores (RSSs) for each cell type. The activity of the regulators was quantified using the AUCell module of pySCENIC, with activity scores represented as AUC values. Active regulators were identified based on the default thresholds set by AUCell. Genes with an importance score greater than 20 were considered the target genes of PRRX2. Based on the PRRX2 target genes, we performed enrichment analysis on each cell using irGSEA (2.1.5).

### 2.5 Differential expression analysis and functional enrichment analysis

Differential expression analysis was performed between the high and low PRRX2-score groups using the limma package (v 3.56.2) (Ritchie et al., 2015). KEGG pathway enrichment was assessed via the gene set enrichment analysis (GSEA) using the gseKEGG function from the clusterProfiler package (v 4.12.1) with BH FDR correction.

### 2.6 Drug sensitivity analysis

The drug sensitivity analysis was conducted using the calcPhenotype function from the oncoPredict package. The datasets for fitting the model were from the GDSC2 database (https://www.cancerrxgene.org/).

### 2.7 Cell culture and transfection

Human intestinal fibroblasts (HIFs) and human colorectal carcinoma cells (HCT116) were revived, centrifuged, and resuspended in complete DMEM supplemented with 10% fetal bovine serum (FBS) and 1% penicillin–streptomycin. Cells were maintained at 37 ℃ in a humidified incubator with 5% CO_2_ and subcultured every 3 days at a 1:3 split ratio. For gene silencing experiments, PRRX2-targeting siRNA (50 μM) was transfected using Lipofectamine™ 3000 according to the manufacturer’s protocol. Cells were collected 24 h post-transfection for downstream analyses.

### 2.8 RNA extraction, reverse transcription, and qPCR

Total RNA was isolated from cultured cells using TRIzol reagent following the manufacturer’s protocol. RNA purity and concentration were assessed using spectrophotometry. First-strand cDNA was synthesized using the ReverTra Ace qPCR RT Kit, and quantitative real-time PCR was carried out with the SYBR Green qPCR Master Mix on an Applied Biosystems 7500 Real-Time PCR System. The primer sequences used were as follows: PRRX2 (Forward: 5′- GCACCACGTTCAACAGCAG-3′, Reverse: 5′- TCCTTGGCCTTGAGACGGA-3′), TGFB1 (Forward: 5′- GGCCAGATCCTGTCCAAGC-3′, Reverse: 5′- GTGGGTTTCCACCATTAGCAC-3′), NGF (Forward: 5′- GGCAGACCCGCAACATTACT-3′, Reverse: 5′- CACCACCGACCTCGAAGTC-3′), β-actin (Forward: 5′- CATGTACGTTGCTATCCAGGC-3′, Reverse: 5′- CTCCTTAATGTCACGCACGAT-3′), siPRRX2 (5′- CCGTCTCAAGGCCAAGGAGTT-3′), and siCtrl (5′- TTCTCCGAACGTGTCACGT-3′). Gene expression was calculated using the 2^−^ΔΔCt method, with β-actin as the internal control. Each qPCR experiment was repeated thrice, and the differences were calculated.

### 2.9 Cell proliferation assay (CCK-8)

HIFs and HCT116 cells were seeded into 96-well plates and transfected; then, they were incubated for 24, 48, 72, and 96 h. At each time point, 10 μL of CCK-8 solution was added per well, followed by a 2-h incubation at 37 ℃, and each assay was repeated thrice. Cell viability was assessed by measuring absorbance at 450 nm using a microplate reader.

### 2.10 Colony formation assay

Transfected HIFs and HCT116 cells were seeded into 6-well plates (1,000 cells per well) and cultured for 14 days. Cells were fixed with 4% paraformaldehyde and stained with crystal violet, and colonies (containing >50 cells) were counted under a microscope.

### 2.11 Transwell migration and invasion assays

HCT116 cells were digested, washed, and resuspended in serum-free medium at 4 × 10^6^ cells/mL. An amount of 500 μL of HIF cells (4 × 10^6^ cells/mL) were seeded per well in 24-well plates. Matrigel-coated Transwell inserts (8.0-μm pore) were placed to allow direct contact with HIFs. Then, 200 μL of HCT116 cells (8 × 10^5^) were added to the upper chamber. After 24 h incubation, non-migrated cells were removed, and invaded cells on the lower membrane surface were fixed (methanol), stained (crystal violet), and quantified under a microscope (100×), analyzing three representative fields per insert. GraphPad Prism was used to draw graphs and to count significance.

### 2.12 Statistical analysis

All analyses were conducted using R version 4.3.1. Survival (v 3.5–7) [62] and Survminer (v 0.4.9) [63] packages were utilized for survival analysis. Kaplan–Meier curves were used to compare survival differences, and Cox proportional hazards regression was utilized to assess survival rates. Statistical significance was determined by the Wilcoxon’s rank-sum test, followed by multiple testing correction using the Holm method. The differences in qPCR expression and cell number were analyzed by t-tests. Differential gene expression between the control and knockdown groups was analyzed using t-tests, with paired comparisons analyzed using the Wilcoxon signed-rank test. Enrichment analysis of drug–target pathways was performed using hypergeometric testing, with p-values adjusted using the BH method.

## 3 Results

### 3.1 Fibroblasts are associated with perineural invasion in colorectal cancer

**FIGURE 1:**
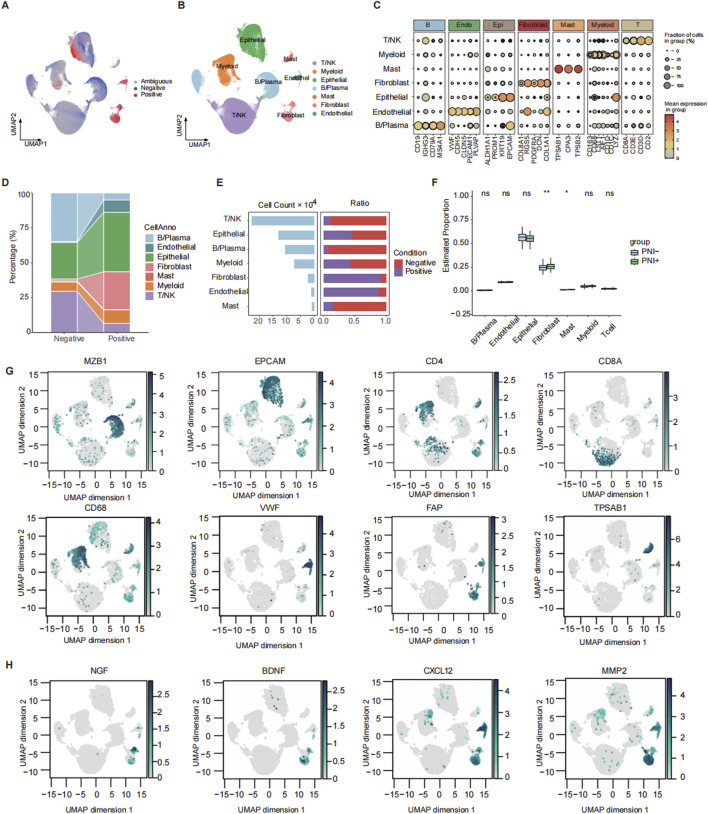
Mapping and annotation of perineural invasion in single-cell data. **(A)** Scissor mapping results of colorectal cancer samples, where positive represents the PNI(+) cells and negative represents the PNI(−) cells. **(B)** UMAP plot of total cell annotation of merged colorectal cancer single-cell data, where different colors represent different cell types. **(C)** Bubble plot of the expression of marker genes for each annotated cell type. **(D)** Sankey plot of the distribution of PNI groups across different cell types. **(E)** Bar plot showing the number of cells in each subtype and corresponding proportions with PNI+ and PNI-. **(F)** Boxplot comparing the deconvoluted infiltration scores of each subtype between patients with or without PNI. **(G)** Feature plot showing the expression of representative marker genes for each subpopulation. **(H)** Feature plot showing the expression of PNI-related factors. ns: p > 0.05; *: p < 0.05; **: p < 0.01.

We integrated 20 CRC single-cell transcriptome datasets and obtained 575,768 high-quality cells via the Seurat integration algorithm. We mapped bulk RNA-seq data with PNI tags from the TCGA CRC cohort (Supplementary Table S5) to all scRNA-seq data by using the Scissor method. Finally, a total of 45,789 PNI-positive (PNI+, 33.5%) and 90,744 PNI-negative (PNI-, 66.5%) cells were identified (Figure 1A; Supplementary Figure S1A, B). In the study, we classified all cells into seven major cell types (Figures 1B, C, G) based on the canonical marker gene. Subsequently, we calculated the silhouette widths for representative cells across each cell type based on the Harmony-integrated embedding (Supplementary Figure S1C), which showed moderate overall cluster separation with a global average silhouette score of 0.439. After comparing the number and proportion of each cell type in the PNI+/PNI- groups, we found that the proportion of fibroblasts and epithelial cells was significantly higher in the PNI+ group (Figure 1D).

To provide further confirmation of the importance of CAF in promoting PNI, we investigated the function of CAF at different levels. The results demonstrated that the proportion of CAF was the greatest in the PNI+ group at the single-cell RNA level (Figure 1E). Deconvolution analysis of bulk RNA from TCGA revealed that the proportion of CAF was higher in the PNI+ population and significantly different from that in the PNI- patients (Figure 1F). The single-cell RNA results were highly consistent with the bulk RNA data. In addition, we analyzed the expression of several PNI-promoting factors that have been reported to be present in CAF. These factors include neurotrophic factors (e.g., NGF and BDNF) (Xu et al., 2019), chemokines (e.g., CXCL12) (Xu et al., 2019), and extracellular matrix remodeling enzymes (e.g., MMPs) (Chen et al., 2023; Tassone et al., 2022). In CRC, we observed high expressions of NGF, BDNF, and MMP2 in fibroblast subpopulations (Figure 1H). This finding suggests the potential for a similar signaling mechanism underlying perineural infiltration in CRC.

### 3.2 MMP2+myCAF promotes perineural invasion and is the biomarker of prognosis

**FIGURE 2:**
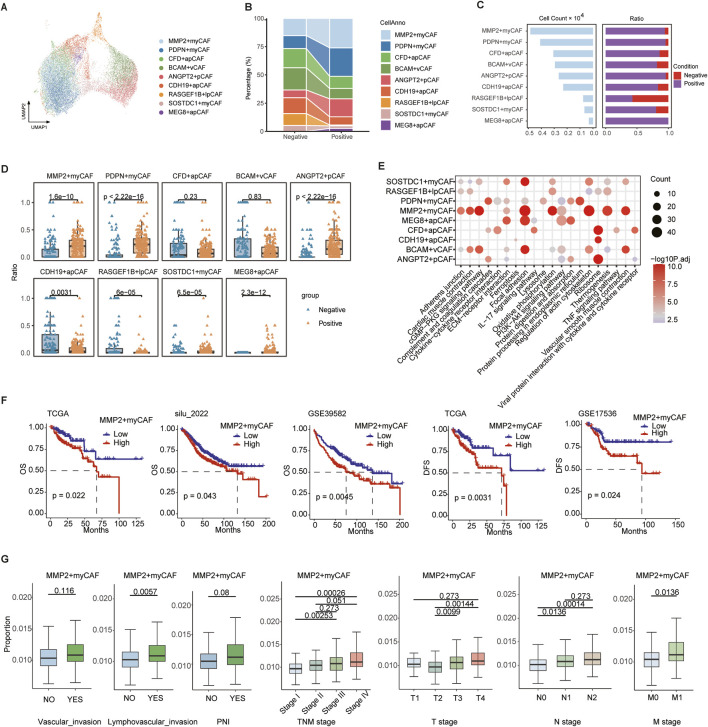
Heterogeneity of fibroblast cells. **(A)** UMAP map of fibroblast cells, with different colors representing distinct subsets. **(B)** Sankey plot showing variation in the proportions of different subtypes between PNI-positive (PNI+) and PNI-negative (PNI-) groups. **(C)** Bar plot showing the number of cells in each subpopulation and corresponding proportions with PNI+ and PNI-. **(D)** Boxplot of nine CAF subpopulation proportions across single-cell samples. **(E)** Bubble plot of the top 10 KEGG pathway enrichment for marker genes of nine CAF subpopulations. **(F)** Survival analysis of MMP2+myCAF infiltration scores: overall survival in TCGA, silu_2022, and GSE39582 cohorts (left three panels); disease-free survival in TCGA and GSE17536 (right two panels). **(G)** Boxplots comparing MMP2+myCAF infiltration scores between patients with or without vascular invasion, lymphovascular invasion, and PNI, and across patients with different TNM stages. Statistical significance was evaluated using the Wilcoxon rank-sum test, with multiple testing correction performed using the Holm method.

Fibroblasts were significantly enriched in the PNI+ group and were further classified into nine subsets with functional heterogeneity (Figure 2A). In addition, MMP2+myCAF, PDPN + myCAF, and ANGPT2+pCAF were significantly higher in the PNI+ group (Figures 2B–D), suggesting that these subsets may play a critical role in the remodeling of the tumor microenvironment associated with neural invasion. A previous study demonstrated that MMP2 was capable of degrading collagen surrounding nerve bundles by facilitating the dissemination of cancer cells within the perineural space in oral cavity squamous cell carcinoma (Tassone et al., 2022).

We identified the highly expressed genes in each subgroup using the FindAllMarkers function and performed KEGG pathway enrichment analysis. The MMP2+myCAF subpopulation was found to be significantly enriched in the ECM–receptor interaction, vascular smooth muscle contraction, and focal adhesion pathway, suggesting that this subset may promote tumor cell infiltration and metastasis by remodeling the extracellular matrix (Figure 2E). The ANGPT2+pCAF subpopulation demonstrated enrichment in the PI3K–Akt signaling pathway and complement and coagulation cascades, indicating its potential involvement in cell survival, proliferation, and metabolism, along with its influence on the local inflammatory response and immune modulation. The PDPN + myCAF subgroup was found to be associated with endoplasmic reticulum protein processing pathways, including protein processing in the endoplasmic reticulum.

The BayesPrism algorithm was employed to deconvolve the aforementioned three CAF subgroups into TCGA and three independent CRC bulk RNA-seq datasets (GSE39582, GSE17536, and silu_2022), assessing their clinical relevance. The infiltration score of MMP2+myCAF was found to be strongly associated with poor patient prognosis in all four datasets. Patients with high infiltration had significantly worse overall survival (OS) and disease-free survival (DFS) scores (Figure 2F). Further clinical stratification analysis of the TCGA cohort revealed that the degree of MMP2+myCAF infiltration increased with increasing clinical stage, and it was considerably higher in the T3-4, N1-2, and M1 groups than in the earlier group (Figure 2G).

In summary, MMP2+myCAF is closely associated with the neuroinvasive phenotype and significantly affects the survival prognosis of patients.

### 3.3 PRRX2 drives perineural invasion *via* TGF-β signaling in MMP2+myCAF

**FIGURE 3:**
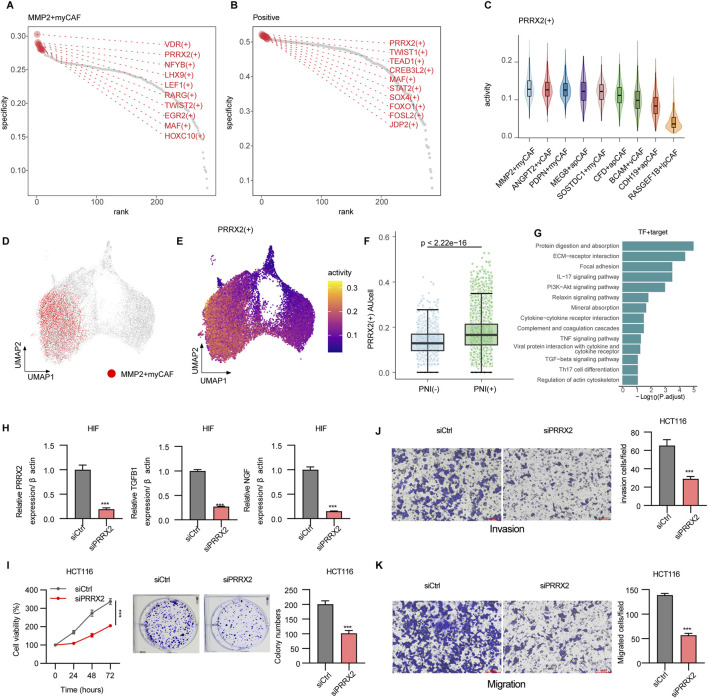
Mechanisms driving perineural invasion in fibroblasts. **(A)** Rank points of the top 10 TFs specifically enriched in the MMP2+myCAF groups. **(B)** Rank points of the top 10 TFs specifically enriched in PNI+ groups. **(C)** Violin plots of PRRX2+ regulon activities across CAF subpopulations. **(D)** DimPlot of all CAF cells, with MMP2+myCAF shown in red. **(E)** Feature plot showing the regulon activity of PRRX2+ regulon. **(F)** Boxplot showing the PRRX2+ regulon activity scores calculated using AUCell. Wilcoxon’s test was used to calculate the statistical significance between PNI (+) vs. PNI (−). **(G)** Bar plot showing the enriched KEGG pathway of PRRX2+ targets. **(H)** PRRX2 knockdown assay in HIF cell line. **(I)** CCK8 and colony formation assay showing inhibition upon PRRX2 knockdown in the HCT116 cell line. Transwell assay demonstrating reduced invasion **(J)** and migration **(K)** after PRRX2 knockdown. Statistical tests were performed using t-test. *: p < 0.05, **: p < 0.01, and ***: p < 0.001.

To explore the key regulatory factors in MMP2+myCAF, we utilized pySCENIC to evaluate the expression levels of transcription factors and their activities in the regulatory network. Using the RSS analysis, MMP2+myCAF type-specific regulons (Figure 3A) and PNI+ group-specific regulons (Figure 3B) were identified. Importantly, the PRRX2 regulon was significantly shared between the two different groups. The expression analysis revealed that PRRX2 was significantly highly expressed and most active in MMP2+myCAF (Figures 3C–E). Furthermore, PRRX2 and its target genes exhibited significantly higher AUCell values in PNI+ samples than in PNI- samples (Figure 3F; Supplementary Table S6), thereby offering additional evidence in support of its hypothesized driving role in the development of PNI. PRRX2 targets showed significant enrichment in the TGF-β signaling pathway and the ECM–receptor interaction pathway, suggesting that PRRX2 may play a role in the invasive behavior of tumor cells in the tumor microenvironment (Figure 3G).

PRRX2 gene knockout *in vitro* resulted in decreased expression of TGF-β1 and NGF, indicating that PRRX2 may promote nerve invasion through the downstream effectors (Figure 3H). The CCK-8 cell proliferation assay revealed a substantial decrease in the proliferation capacity of tumor-associated fibroblasts following PRRX2 knockdown (Figure 3I). Moreover, the number of cell clones was significantly reduced after PRRX2 knockdown in HIFs and co-culture with the CRC cell line HCT116 by the clone formation assay (Figure 3I). Furthermore, the Transwell invasion and migration (Figure 3J, K; Supplementary Figure S2) assays further confirmed that PRRX2 knockdown significantly inhibited the cell invasiveness and migration ability, suggesting its multiple mechanistic roles in promoting tumor progression and PNI.

Overall, PRRX2 played a core regulatory role in MMP2+myCAF and a key role in promoting the PNI by regulating TGF-β-related pathways.

### 3.4 High PRRX2-score predicts poor prognosis and correlates with malignant features

**FIGURE 4:**
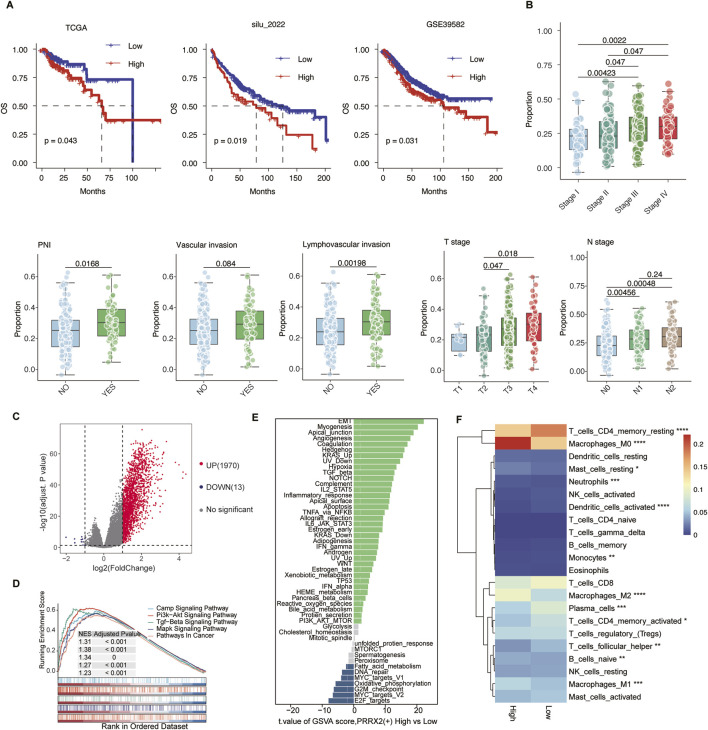
PRRX2 score may predict patient prognosis and is significantly associated with malignant clinical characteristics. **(A)** Kaplan–Meier survival curves demonstrating the relationship between the PRRX2 score and patient overall survival in the TCGA, silu_2022, and GSE39582 cohorts. **(B)** Boxplot comparing the PRRX2 scores between patients with or without vascular invasion, lymphovascular invasion, and PNI, and across patients with different TNM stages. Statistical significance was evaluated using the Wilcoxon rank-sum test, with multiple testing correction performed using the Holm method. **(C)** Volcano plots showing the differentially expressed genes between high and low PRRX2-score groups. **(D)** Gene-set enrichment analysis (GSEA) showing the enrichment in the KEGG pathway for the differentially expressed genes. **(E)** Differential pathway enrichment analysis based on GSVA between PRRX2-high and PRRX2-low groups. The bar plot displays pathways ranked by the t-value of the GSVA score (high vs. low PRRX2 score). **(F)** Heatmap showing the infiltration level of 22 immune-cell types in high and low PRRX2-score groups. *: p < 0.05; **: p < 0.01; ***: p < 0.001; ****: p < 0.0001.

We constructed a PRRX2 score using the ssGSEA method in four datasets of the CRC transcriptome to predict the prognosis. The Kaplan–Meier survival analysis was performed after categorizing patients into high- and low-expression groups according to the median PRRX2 score. The high-score patients had significantly poorer OS and DFS (both p < 0.05) (Figure 4A). Further analysis revealed a significant positive correlation between the PRRX2 score and tumor progression (Figure 4B). The PRRX2 score exhibited a progressive increase with the progression of the clinical stage, and it was also significantly upregulated in patients with more advanced tumor infiltration depth (T-stage) and lymph node metastasis (N-stage). Notably, the PRRX2 score was significantly higher in patients with LVI and PNI, indicating that this characteristic score can effectively reflect the aggressive biological behavior of the tumor (Figure 4B).

The samples were grouped into high and low PRRX2 score groups based on the median value. Subsequent differential expression analysis uncovered several genes that were significantly upregulated in the high-score subgroup (Figure 4C). These genes were enriched in the PI3K-AKT and TGF-β pathways, as shown by KEGG pathway enrichment analysis (Figure 4D). GSVA was used to compare the cancer hallmark enrichment between high and low PRRX2 score groups, and the results showed that the PRRX2-high group was enriched in EMT, myogenesis, and other pathways (Figure 4E). The results demonstrated that the PRRX2 regulon may promote tumor progression, proliferation, and invasive behavior by regulating these key cancer-related pathways in MMP2+myCAF. We then compared immune infiltration between the two groups. Patients with elevated PRRX2 scores exhibited an increase in immunosuppressive cell populations, including natural killer cells and regulatory T cells (Figure 4F). These findings suggest that PRRX2 may play a synergistic role in immune escape and tumor microenvironment remodeling to evade immunosurveillance.

In summary, the PRRX2 score could be used as an independent predictor of prognosis in CRC (Supplementary Figure S3). High PRRX2 scores were associated with a stronger immunosuppressive immune microenvironment and promotion of EMT, which was the key reason for PNI.

### 3.5 PRRX2 score predicts immunotherapy outcomes and promising treatment agents

**FIGURE 5:**
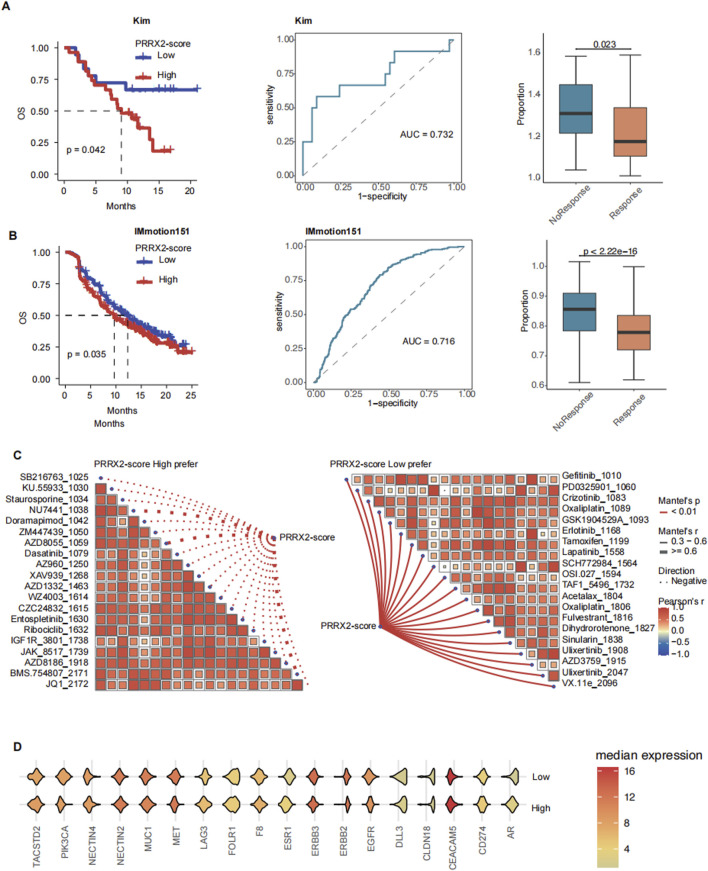
PRRX2 score can predict immunotherapy outcomes and promising drug agents. Kaplan–Meier survival curves demonstrating the relationship between the PRRX2 score and patient overall survival in **(A)** Kim and **(B)** IMmotion151 cohorts (left panel). Receiver operating characteristic (ROC) curve for PRRX2 score in predicting immunotherapy response in **(A)** Kim and **(B)** IMmotion151 immunotherapy cohorts (middle panel). Boxplot comparing the PRRX2 score between immunotherapy response and non-response groups of **(A)** Kim and **(B)** IMmotion151 cohorts (right panel). **(C)** Spearman correlation analysis between PRRX2-score high prefer (left) and PRRX2-score low prefer (right) scores. **(D)** Violin plot of the expression of ADC drug target genes across high PRRX2-score and low PRRX2-score groups.

The PRRX2 score could effectively predict immunotherapy response and prognosis. In this work, we found that patients with low PRRX2 scores had a good prognosis for immunotherapy (Kim cohort: p = 0.042; IMmotion151 cohort: p = 0.035) in several immunotherapy cohorts (Figures 5A,B; Supplementary Table S7). In the two immunotherapy cohorts, the area under the curve (AUC) for distinguishing immunotherapy response based on the PRRX2 score was higher than 0.7 (Kim cohort: AUC = 0.732; IMmotion151 cohort: AUC = 0.716), and the PRRX2 score of the non-response group was higher than that of the response group (Figures 5A, B).

To improve patient benefit, we continued to explore alternative treatment strategies. We performed drug sensitivity analysis and identified several inhibitors that may be effective in patients with low PRRX2 scores, such as EGFR inhibitors and MEK/ERK pathway inhibitors (Figure 5C). Patients with elevated PRRX2 scores were sensitized to several targeted chemotherapy agents (CZC24832, KU.55933, staurosporine, *etc*.) and ADCs (ZM447439, BMS.754807, *etc*.) (Figure 5C). These findings indicated that potential alternative options were available for patients with poor immunotherapy outcomes. Subsequently, an analysis was conducted on the expression levels of ADC-related targets. The results revealed that genes such as PIK3CA, MUC1, and MET were highly expressed in the high PRRX2-score population (Figure 5E). This finding indicated that these ADCs may also have a helpful effect in treating patients with high PRRX2 scores, which provides a new approach for the precision treatment for these patients with CRC.

In summary, the PRRX2 score can be used to predict the response to immunotherapy and survival. This model provides an important adjuvant role for personalized precision therapy. For patients with high PRRX2 scores, potentially beneficial combination therapy options such as ADC and targeted therapy can be used as alternatives.

## 4 Discussion

Although the mechanism of action of PNI has been investigated in some solid tumors, the underlying molecular mechanism in CRC is not completely understood (Knijn et al., 2016; Cao et al., 2020). In our study, we integrated 20 single-cell transcriptome datasets to explore the cellular and molecular basis of PNI in CRC. We revealed a significant enrichment of CAF in PNI-positive cells. Among nine CAF subtypes, MMP2+myCAF exhibited the most significant accumulation. In addition, MMP2+myCAF was found to have a strong correlation with advanced clinical stages and a poor prognosis.

We identified PRRX2 as a critical transcription factor in MMP2+myCAF utilizing a regulatory network-based analysis. PRRX2 enhanced the PNI capacity of CAFs by activating the TGF-β signaling pathway. Knockdown of PRRX2 resulted in significant downregulation of TGFB1 and NGF, which suggested that PRRX2 may promote PNI through these downstream effectors. In addition, co-culture experiment results demonstrated that PRRX2 knockdown significantly inhibited the proliferation and invasive migration ability of cancer-associated fibroblasts. Conclusively, our results confirmed that PRRX2 may promote PNI through multiple molecular mechanisms.

To further explore the key regulators that promote PNI in MMP2+myCAF, we performed a regulatory network analysis and focused on the action of the PRRX2 regulon. The PRRX2 score was effective in differentiating patient prognosis, and high scores were significantly associated with advanced clinical stages, vascular/lymphatic infiltration, and PNI. A high PRRX2 score presented increased Treg and NK cell infiltration. Moreover, immunotherapy prognosis and response could also be significantly predicted by the PRRX2 score. High PRRX2-score patients may benefit from combination therapy with targeted agents, such as AZD8055 and NU7441, or ADC drugs, such as PIK3CA, MUC1, and MET.

However, there are some limitations to our study. First, this study lacks *in vivo* experimental validation, such as mouse experiments. Additionally, subsequent studies will need to validate the function of the PRRX2 score in large clinical cohorts.

## 5 Conclusion

In conclusion, our study identified MMP2+myCAF as a significant CAF subpopulation of PNI+ cells. In the MMP2+myCAF subpopulation, PRRX2 was a typical regulator that can promote PNI through the TGF-β signaling pathway. In the future, the integration of single-cell multi-omics may elucidate how PRRX2+CAF cooperates with other microenvironmental components to promote PNI and provide a theoretical basis for precision therapy.

## Data Availability

The original contributions presented in the study are included in the article/Supplementary Material further inquiries can be directed to the corresponding authors.

## Abbreviations

PNI, perineural invasion; CRC, colorectal cancer; CAF, cancer-associated fibroblasts; myCAFs, myofibroblastic cancer-associated fibroblasts; scRNA-seq, single-cell RNA sequencing; TCGA, The Cancer Genome Atlas; KEGG, Kyoto Encyclopedia of Genes and Genomes; TFs, transcription factors; RSS, regulon specificity scores; HIFs, human intestinal fibroblasts; HCT116, human colorectal carcinoma cells; OS, overall survival; DFS, disease-free survival; T-stage, tumor infiltration depth; N-stage, lymph node metastasis; ICBs, immune checkpoint inhibitors; ADCs, antibody-coupled drugs.

## References

[B1] Becker W. R. Nevins S. A. Chen D. C. Chiu R. Horning A. M. Guha T. K. (2022). Single-cell analyses define a continuum of cell state and composition changes in the malignant transformation of polyps to colorectal cancer. Nat. Genet. 54, 985–995. 10.1038/s41588-022-01088-x 35726067 PMC9279149

[B2] Berlin C. Mauerer B. Cauchy P. Luenstedt J. Sankowski R. Marx L. (2023). Single-cell deconvolution reveals high lineage- and location-dependent heterogeneity in mesenchymal multivisceral stage 4 colorectal cancer. J. Clin. Invest 134, e169576. 10.1172/JCI169576 38153787 PMC10904044

[B3] Cao Y. Deng S. Yan L. Gu J. Li J. Wu K. (2020). Perineural invasion is associated with poor prognosis of colorectal cancer: a retrospective cohort study. Int. J. Colorectal Dis. 35, 1067–1075. 10.1007/s00384-020-03566-2 32179991

[B4] Che L. H. Liu J. W. Huo J. P. Luo R. Xu R. M. He C. (2021). A single-cell atlas of liver metastases of colorectal cancer reveals reprogramming of the tumor microenvironment in response to preoperative chemotherapy. Cell Discov. 7, 80. 10.1038/s41421-021-00312-y 34489408 PMC8421363

[B5] Chen Z. Fang Y. Jiang W. (2023). Important cells and factors from tumor microenvironment participated in perineural invasion. Cancers (Basel) 15, 1360. 10.3390/cancers15051360 36900158 PMC10000249

[B6] Chen Y. Wang D. Li Y. Qi L. Si W. Bo Y. (2024). Spatiotemporal single-cell analysis decodes cellular dynamics underlying different responses to immunotherapy in colorectal cancer. Cancer Cell 42, 1268–1285.e7. 10.1016/j.ccell.2024.06.009 38981439

[B7] Chu T. Wang Z. Pe'er D. Danko C. G. (2022). Cell type and gene expression deconvolution with BayesPrism enables Bayesian integrative analysis across bulk and single-cell RNA sequencing in oncology. Nat. Cancer 3, 505–517. 10.1038/s43018-022-00356-3 35469013 PMC9046084

[B8] Cienfuegos J. A. Martinez P. Baixauli J. Beorlegui C. Rosenstone S. Sola J. J. (2017). Perineural invasion is a major prognostic and predictive factor of response to adjuvant chemotherapy in stage I-II colon cancer. Ann. Surg. Oncol. 24, 1077–1084. 10.1245/s10434-016-5561-0 27624582

[B9] Gao J. Aksoy B. A. Dogrusoz U. Dresdner G. Gross B. Sumer S. O. (2013). Integrative analysis of complex cancer genomics and clinical profiles using the cBioPortal. Sci. Signal 6, pl1. 10.1126/scisignal.2004088 23550210 PMC4160307

[B10] Guo W. Zhang C. Wang X. Dou D. Chen D. Li J. (2022). Resolving the difference between left-sided and right-sided colorectal cancer by single-cell sequencing. JCI Insight 7, e152616. 10.1172/jci.insight.152616 34793335 PMC8765049

[B11] Hao Y. Hao S. Andersen-Nissen E. Mauck W. M. 3rd Zheng S. Butler A. (2021). Integrated analysis of multimodal single-cell data. Cell 184, 3573–3587.e29. 10.1016/j.cell.2021.04.048 34062119 PMC8238499

[B12] Hsu W. H. LaBella K. A. Lin Y. Xu P. Lee R. Hsieh C. E. (2023). Oncogenic KRAS drives lipofibrogenesis to promote angiogenesis and colon cancer progression. Cancer Discov. 13, 2652–2673. 10.1158/2159-8290.CD-22-1467 37768068 PMC10807546

[B13] Kang J. H. Son I. T. Kim B. C. Park J. H. Kim J. Y. Kim J. W. (2022). Recurrence-free survival outcomes based on novel classification combining lymphovascular invasion, perineural invasion, and T4 status in stage II-III colon cancer. Cancer Manag. Res. 14, 2031–2040. 10.2147/CMAR.S358939 35757161 PMC9231686

[B14] Khaliq A. M. Erdogan C. Kurt Z. Turgut S. S. Grunvald M. W. Rand T. (2022). Refining colorectal cancer classification and clinical stratification through a single-cell atlas. Genome Biol. 23, 113. 10.1186/s13059-022-02677-z 35538548 PMC9092724

[B15] Kim S. T. Cristescu R. Bass A. J. Kim K. M. Odegaard J. I. Kim K. (2018). Comprehensive molecular characterization of clinical responses to PD-1 inhibition in metastatic gastric cancer. Nat. Med. 24, 1449–1458. 10.1038/s41591-018-0101-z 30013197

[B16] Knijn N. Mogk S. C. Teerenstra S. Simmer F. Nagtegaal I. D. (2016). Perineural invasion is a strong prognostic factor in colorectal cancer: a systematic review. Am. J. Surg. Pathol. 40, 103–112. 10.1097/PAS.0000000000000518 26426380

[B17] Lee H. O. Hong Y. Etlioglu H. E. Cho Y. B. Pomella V. Van den Bosch B. (2020). Lineage-dependent gene expression programs influence the immune landscape of colorectal cancer. Nat. Genet. 52, 594–603. 10.1038/s41588-020-0636-z 32451460

[B18] Li J. Kang R. Tang D. (2021). Cellular and molecular mechanisms of perineural invasion of pancreatic ductal adenocarcinoma. Cancer Commun. (Lond) 41, 642–660. 10.1002/cac2.12188 34264020 PMC8360640

[B19] Li J. Mei S. Zhou S. Zhao F. Liu Q. (2022). Perineural invasion is a prognostic factor in stage II colorectal cancer but not a treatment indicator for traditional chemotherapy: a retrospective cohort study. J. Gastrointest. Oncol. 13, 710–721. 10.21037/jgo-22-277 35557585 PMC9086063

[B20] Liu X. Wang X. Yang Q. Luo L. Liu Z. Ren X. (2024). Th17 cells secrete TWEAK to Trigger epithelial-mesenchymal Transition and promote colorectal cancer liver metastasis. Cancer Res. 84, 1352–1371. 10.1158/0008-5472.CAN-23-2123 38335276

[B21] Luo H. Xia X. Huang L. B. An H. Cao M. Kim G. D. (2022). Pan-cancer single-cell analysis reveals the heterogeneity and plasticity of cancer-associated fibroblasts in the tumor microenvironment. Nat. Commun. 13, 6619. 10.1038/s41467-022-34395-2 36333338 PMC9636408

[B22] Marisa L. de Reynies A. Duval A. Selves J. Gaub M. P. Vescovo L. (2013). Gene expression classification of colon cancer into molecular subtypes: characterization, validation, and prognostic value. PLoS Med. 10, e1001453. 10.1371/journal.pmed.1001453 23700391 PMC3660251

[B23] Motzer R. J. Powles T. Atkins M. B. Escudier B. McDermott D. F. Alekseev B. Y. (2022). Final overall survival and molecular analysis in IMmotion151, a phase 3 trial comparing atezolizumab plus bevacizumab vs sunitinib in patients with previously untreated metastatic renal cell carcinoma. JAMA Oncol. 8, 275–280. 10.1001/jamaoncol.2021.5981 34940781 PMC8855230

[B24] Pelka K. Hofree M. Chen J. H. Sarkizova S. Pirl J. D. Jorgji V. (2021). Spatially organized multicellular immune hubs in human colorectal cancer. Cell 184, 4734–4752.e20. 10.1016/j.cell.2021.08.003 34450029 PMC8772395

[B25] Qaderi S. M. Galjart B. Verhoef C. Slooter G. D. Koopman M. Verhoeven R. H. A. (2021). Disease recurrence after colorectal cancer surgery in the modern era: a population-based study. Int. J. Colorectal Dis. 36, 2399–2410. 10.1007/s00384-021-03914-w 33813606 PMC8505312

[B26] Que Y. Wu R. Li H. Lu J. (2024). A prediction nomogram for perineural invasion in colorectal cancer patients: a retrospective study. BMC Surg. 24, 80. 10.1186/s12893-024-02364-9 38439014 PMC10913563

[B27] Ritchie M. E. Phipson B. Wu D. Hu Y. Law C. W. Shi W. (2015). Limma powers differential expression analyses for RNA-sequencing and microarray studies. Nucleic Acids Res. 43, e47. 10.1093/nar/gkv007 25605792 PMC4402510

[B28] Roelands J. Kuppen P. J. K. Ahmed E. I. Mall R. Masoodi T. Singh P. (2023). An integrated tumor, immune and microbiome atlas of colon cancer. Nat. Med. 29, 1273–1286. 10.1038/s41591-023-02324-5 37202560 PMC10202816

[B29] Smith J. J. Deane N. G. Wu F. Merchant N. B. Zhang B. Jiang A. (2010). Experimentally derived metastasis gene expression profile predicts recurrence and death in patients with colon cancer. Gastroenterology 138, 958–968. 10.1053/j.gastro.2009.11.005 19914252 PMC3388775

[B30] Sui Q. Zhang X. Chen C. Tang J. Yu J. Li W. (2022). Inflammation promotes resistance to immune checkpoint inhibitors in high microsatellite instability colorectal cancer. Nat. Commun. 13, 7316. 10.1038/s41467-022-35096-6 36443332 PMC9705377

[B31] Tassone P. Caruso C. White M. Tavares Dos Santos H. Galloway T. Dooley L. (2022). The role of matrixmetalloproteinase-2 expression by fibroblasts in perineural invasion by oral cavity squamous cell carcinoma. Oral Oncol. 132, 106002. 10.1016/j.oraloncology.2022.106002 35779484

[B32] Uhlitz F. Bischoff P. Peidli S. Sieber A. Trinks A. Luthen M. (2021). Mitogen-activated protein kinase activity drives cell trajectories in colorectal cancer. EMBO Mol. Med. 13, e14123. 10.15252/emmm.202114123 34409732 PMC8495451

[B33] Van de Sande B. Flerin C. Davie K. De Waegeneer M. Hulselmans G. Aibar S. (2020). A scalable SCENIC workflow for single-cell gene regulatory network analysis. Nat. Protoc. 15, 2247–2276. 10.1038/s41596-020-0336-2 32561888

[B34] Wang H. Gong P. Chen T. Gao S. Wu Z. Wang X. (2021). Colorectal cancer stem cell States uncovered by simultaneous single-cell analysis of transcriptome and telomeres. Adv. Sci. (Weinh) 8, 2004320. 10.1002/advs.202004320 33898197 PMC8061397

[B35] Wang J. He S. Cao M. Teng Y. Li Q. Tan N. (2024). Global, regional, and national burden of colorectal cancer, 1990-2021: an analysis from global burden of disease study 2021. Chin. J. Cancer Res. 36, 752–767. 10.21147/j.issn.1000-9604.2024.06.12 39802900 PMC11724172

[B36] Xu Z. Zheng X. Zheng J. (2019). Tumor-derived exosomes educate fibroblasts to promote salivary adenoid cystic carcinoma metastasis via NGF-NTRK1 pathway. Oncol. Lett. 18, 4082–4091. 10.3892/ol.2019.10740 31516608 PMC6733016

[B37] Yang M. W. Tao L. Y. Jiang Y. S. Yang J. Y. Huo Y. M. Liu D. J. (2020). Perineural invasion reprograms the immune microenvironment through cholinergic signaling in pancreatic ductal adenocarcinoma. Cancer Res. 80, 1991–2003. 10.1158/0008-5472.CAN-19-2689 32098780

[B38] Yu G. Wang L. G. Han Y. He Q. Y. (2012). clusterProfiler: an R package for comparing biological themes among gene clusters. OMICS 16, 284–287. 10.1089/omi.2011.0118 22455463 PMC3339379

[B39] Zhang J. Li S. Zhao Y. Ma P. Cao Y. Liu C. (2020a). Cancer-associated fibroblasts promote the migration and invasion of gastric cancer cells via activating IL-17a/JAK2/STAT3 signaling. Ann. Transl. Med. 8, 877. 10.21037/atm-20-4843 32793721 PMC7396760

[B40] Zhang L. Li Z. Skrzypczynska K. M. Fang Q. Zhang W. O'Brien S. A. (2020b). Single-cell analyses inform mechanisms of myeloid-targeted therapies in colon cancer. Cell 181, 442–459. 10.1016/j.cell.2020.03.048 32302573

[B41] Zhang X. He Y. Xie S. Song Y. Huang X. Hu Q. (2025). Cancer-associated fibroblasts interact with Schwann cells for tumor perineural invasion by oral squamous cell carcinoma. Neurosci. Bull. 41, 1003–1020. 10.1007/s12264-025-01364-w 39998796 PMC12158880

[B42] Zheng X. Song J. Yu C. Zhou Z. Liu X. Yu J. (2022). Single-cell transcriptomic profiling unravels the adenoma-initiation role of protein tyrosine kinases during colorectal tumorigenesis. Signal Transduct. Target Ther. 7, 60. 10.1038/s41392-022-00881-8 35221332 PMC8882672

[B43] Zheng S. Hu C. Lin Q. Li T. Li G. Tian Q. (2024). Extracellular vesicle-packaged PIAT from cancer-associated fibroblasts drives neural remodeling by mediating m5C modification in pancreatic cancer mouse models. Sci. Transl. Med. 16, eadi0178. 10.1126/scitranslmed.adi0178 39018369

[B44] Zhou Y. Zhang X. Gao Y. Peng Y. Liu P. Chen Y. (2024). Neuromedin U receptor 1 deletion leads to impaired immunotherapy response and high malignancy in colorectal cancer. iScience 27, 110318. 10.1016/j.isci.2024.110318 39055918 PMC11269305

