# Supplementary figures and images for: Single-cell and bulk RNA-sequencing reveal PRRX2-driven cancer-associated fibroblast-mediated perineural invasion for predicting the immunotherapy outcome in colorectal cancer
Source: Front Cell Dev Biol. 2025 Sep 29;13:1620388. doi: 10.3389/fcell.2025.1620388 (PMC12516196; doi:10.3389/fcell.2025.1620388)

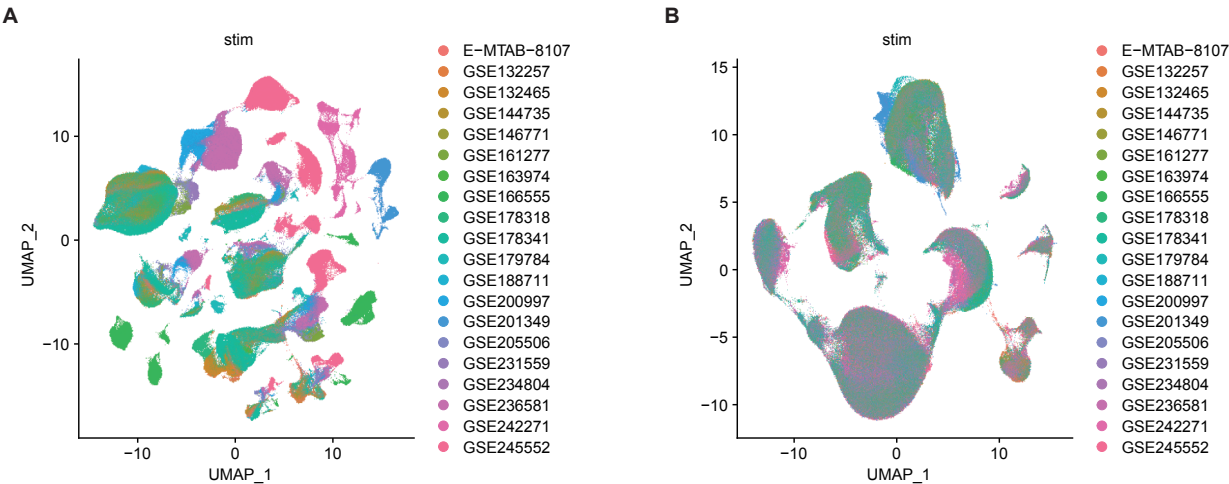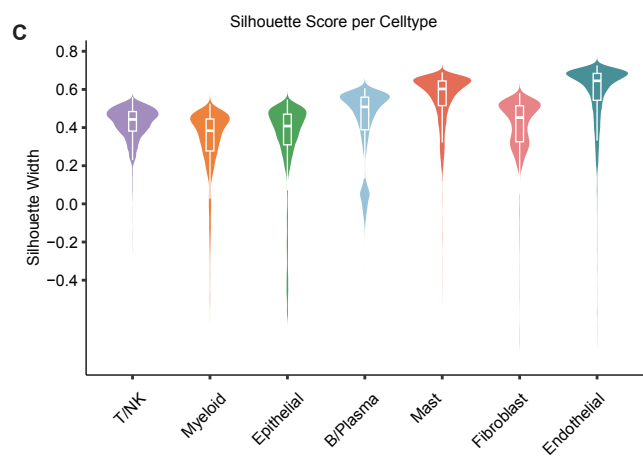

Supplement: Supplementary file 1 [file DataSheet2.pdf]

**A**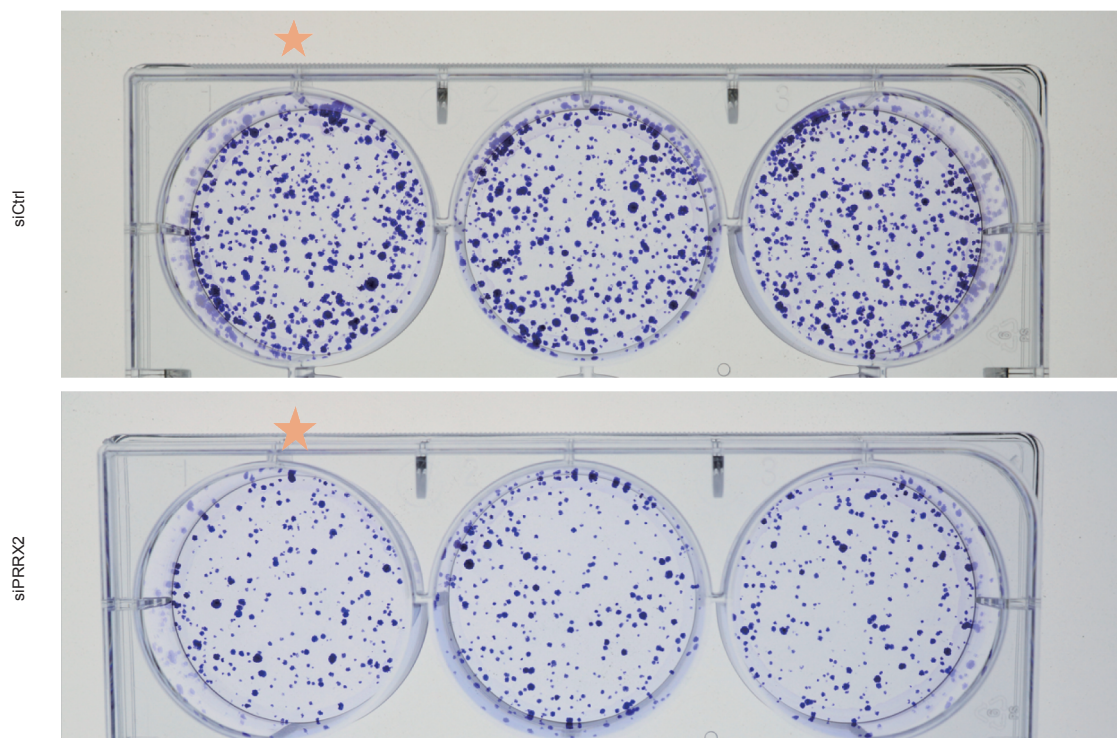**B**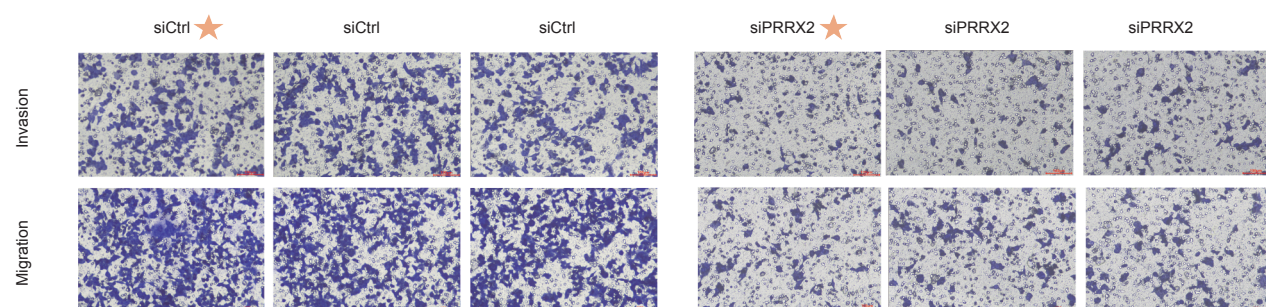

Supplement: Supplementary file 4 [file DataSheet3.pdf]
