# Supplementary material for: Single-cell and bulk RNA-sequencing reveal PRRX2-driven cancer-associated fibroblast-mediated perineural invasion for predicting the immunotherapy outcome in colorectal cancer
Source: Front Cell Dev Biol. 2025 Sep 29;13:1620388. doi: 10.3389/fcell.2025.1620388 (PMC12516196; doi:10.3389/fcell.2025.1620388)

Characteristics	HR (95%CI)	P value
PRRX2	[3.47E+35,1.91E+104]	6.71e−05
MSI(Yes vs No)	[0,Inf]	0.997
KRAS_Mut(Yes vs No)	[0.63,2.97]	0.421
BRAF_Mut(Yes vs No)	[0,Inf]	0.998

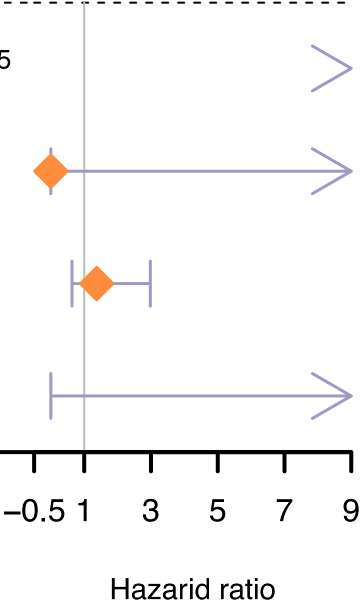

Supplement: Supplementary file 2 [file DataSheet4.pdf]
